# Sortilin inhibition limits secretion-induced progranulin-dependent breast cancer progression and cancer stem cell expansion

**DOI:** 10.1186/s13058-018-1060-5

**Published:** 2018-11-20

**Authors:** Sara Rhost, Éamon Hughes, Hannah Harrison, Svanheidur Rafnsdottir, Hanna Jacobsson, Pernilla Gregersson, Ylva Magnusson, Paul Fitzpatrick, Daniel Andersson, Karoline Berger, Anders Ståhlberg, Göran Landberg

**Affiliations:** 1 Department of Pathology and Genetics, Institute of Biomedicine, Sahlgrenska Cancer Center, University of Gothenburg, Gothenburg, Sweden; 2 Division of Molecular and Clinical Cancer Sciences, Manchester Cancer Research Centre, University of Manchester, Wilmslow Road, Manchester, M20 4QL UK; 3 Shore Lab, Faculty of Life Sciences, University of Manchester, Michael Smith Building, Oxford Road, Manchester, M13 9PT UK; 4 Department of Surgery, Sahlgrenska University Hospital, Gothenburg, Sweden; 5 Institute of Clinical Sciences, Sahlgrenska Academy, Gothenburg, Sweden; 6 Wallenberg Centre for Molecular and Translational Medicine, University of Gothenburg, Gothenburg, Sweden; 7 Department of Clinical Pathology and Genetics, Sahlgrenska University Hospital, 413 45 Gothenburg, Sweden

**Keywords:** Cancer stem cells, Breast cancer, Hypoxia, Secretion, Dedifferentiation, Metastasis, Differentiation

## Abstract

**Background:**

Cancer progression is influenced by genetic aberrations in the cancer cell population as well as by other factors including the microenvironment present within a tumour. Direct interactions between various cell types as well as cellular signalling via secreted cytokines can drive key tumourigenic properties associated with disease progression and treatment resistance. Also, cancer stem cell functions are influenced by the microenvironment. This challenging subset of cells has been linked to malignant properties. Within a screen, using in vivo like growth conditions, we identified progranulin as a highly secreted cytokine affecting cancer stem cells in breast cancer. This cytokine is known to play a role in numerous biological and tumour-related processes including therapy resistance in a range of cancer types.

**Methods:**

Different in vitro and in vivo relevant conditions were used to validate breast cancer stem cell expansion mediated by progranulin and its receptor sortilin. Small interfering ribonucleic acid (siRNA) and pharmacological inhibition of sortilin were used to elucidate the role of sortilin as a functional receptor during progranulin-induced breast cancer stem cell propagation, both in vitro and in vivo, using breast cancer xenograft models*.* In addition, single-cell gene expression profiling as well as a Sox2 reporter breast cancer cell line were used to validate the role of dedifferentiation mediated by progranulin.

**Results:**

In various in vivo-like screening assays, progranulin was identified as a potent cancer stem cell activator, highly secreted in ERα-negative breast cancer as well as in ERα-positive breast cancer under hypoxic adaptation. Progranulin exposure caused dedifferentiation as well as increased proliferation of the cancer stem cell pool, a process that was shown to be dependent on its receptor sortilin. Subcutaneous injections of progranulin or its active domain (GRN A) induced lung metastases in breast cancer xenograft models, supporting a major role for progranulin in cancer progression. Importantly, an orally bioavailable small molecule (AF38469) targeting sortilin, blocked GRN A-induced lung metastases and prevented cancer cell infiltration of the skin.

**Conclusion:**

The collective results suggest that sortilin targeting represents a potential novel breast cancer therapy approach inhibiting tumour progression driven by secretion and microenvironmental influences.

**Electronic supplementary material:**

The online version of this article (10.1186/s13058-018-1060-5) contains supplementary material, which is available to authorized users.

## Background

The presence and specific qualities of cancer stem cells (CSCs) has been described as the major reasons for why many solid tumours, including breast cancer, progress and relapse after treatment. CSCs are resistant to conventional therapy, they are readily motile and have the ability to self-renew, clearly demonstrate that they possess the capacity to drive tumour formation and disease progression [1–5]. Specific targeting of CSC regulation and propagation is therefore an attractive cancer therapeutic approach that could lead to better control of tumour progression and improved patient outcome. Besides, genetic and epigenetic alterations in cancer cells, which in turn influence CSC quantities in a tumour, recent research has been focused on the microenvironmental factors influencing breast cancer stem cells. How cancer cells respond to environmental stress, as well as the potential communication with nearby tumour cells to spread cellular differentiation signals has not yet been fully described, but might be of importance for the understanding of tumour progression as well as offering novel therapeutic targets.

Here we identify the signalling protein progranulin as a secreted CSC modulator, contributing to breast cancer progression in various clinical model systems. Progranulin (encoded by the gene *GRN*), also known as prostate cancer cell-derived growth factor (PCDGF), or granulin/epithelin precursor, is a secreted 88-kDa glycoprotein composed of 7.5 cysteine-rich tandem repeats [6–9]. Further, cleavage of progranulin by proteolytic processing, predominantly by neutrophil elastase and matrix metallopeptidase 12 (MMP12) produces biological active 6-kDa granulin peptide domains [10, 11]. These granulins are named from the N terminal of progranulin to the C terminal as, granulin p, G, F, B, A, C, D, and E, with “p” denoting the half-length “paragranulin” domain [7]. Furthermore, progranulin has been characterised as an autocrine growth factor affecting numerous biological and tumour-related processes, including proliferation, survival, migration, angiogenesis, wound repair and neuroinflammation [6, 12, 13]. Several studies have demonstrated that progranulin plays a role in tumour growth as well as therapy resistance in a range of cancer types including breast cancer, although its exact mechanism of action remains unclear [14, 15]. Recently, the neuronal receptor sortilin has been shown to bind to progranulin and mediate progranulin internalisation via an endocytotic mechanism [16]. Interestingly, sortilin has been associated with metastatic potential in breast cancer [17] and is highly expressed in breast cancer cell lines compared to non-tumorigenic breast epithelial cells [17]. In addition, siRNA knockdown of sortilin inhibited breast cancer cell adhesion, migration and invasion suggesting that sortilin indeed is involved in breast cancer progression [17]. Here, we present novel data that demonstrate how secretion of progranulin induce expansion of CSC cells as well as converts differentiated sortilin-positive cells into a CSC-like state, rendering them more aggressive and metastatic independently of estrogen receptor alpha (ERα) status, suggesting that the progranulin-sortilin communication axis may be an important therapeutic target.

## Methods

### Cell lines and cell culture

MCF7, T47D, MDA-MB 231, CAL-120, MCF10A and MDA-MB 468 were purchased from American Type Culture Collection (ATCC, Manassas, VA, USA). All cells were authenticated by ATCC using short tandem repeat profiling prior to use. Monolayers were grown in Dulbecco modified Eagle’s medium (DMEM) (Lonza, Basel, Switzerland) (10% fetal calf serum (FCS)/2 mmol/L L-glutamine/penstrep), or Roswell Park Memorial Institute (RPMI) medium (Thermo Fisher Scientific, Waltham, MA, USA) (10% FCS/1% sodium pyruvate/2 mmol/L L-glutamine/penstrep). Cells were maintained in a humidified incubator at 37 °C at an atmospheric pressure of 5% (v/v) CO2. All cell lines were validated as mycoplasma-negative using a Mycoplasma PCR kit (Applied Biological Materials, Richmond, BC, Canada) in house.

### Hypoxic cell culture

Cells were incubated for 48 h in the SCI-tiveN hypoxic workstation (Ruskinn, Brigend, US) in 1% O_2_, 5% CO_2_, and 94% N_2_ in a humidified environment at 37 °C. Cells were seeded, cultured, and harvested within the workstation to maintain hypoxia at all times. Confirmation of hypoxic conditions was carried out using western blot analysis of HIF-1α protein expression.

### Peptide treatments

Progranulin (Nordic Biosite, Täby, Sweden), GRN A (Caslo [Lyngby, Denmark] peptide sequence: DVKCDMEVSCPDGYTCCRLQSGAWGCCPFTQAVCCEDHIHCCPAGFTCDT QKGTCE-NH2) and secretory leukocyte protease inhibitor (SPLI) (R&D Systems, Minneapolis, MN, USA) were reconstituted in sterile PBS upon delivery, aliquoted and stored at ˗20 °C. Working stock concentrations were achieved by dilution in culture media. Cell lines were treated with indicated concentrations of peptide for 48 h at 37 °C 5% CO_2_ and 21% O_2_.

### Alamar Blue

Cell viability was determined by an Alamar Blue-based metabolic assay according to the manufacturer’s instructions (Invitrogen, San Diego, CA, USA). At 72 h after treatment with either PBS/DMSO or 3 μg/ml AF38469 with or without 1 μg/ml progranulin (PGRN), Alamar Blue reagent was added to each well and absorbance (ΔOD_570 nm–600 nm_) measured on an automated 96-well spectrophotometer after a defined time period for colour development.

### Mammosphere assay

Mammosphere culturing was carried out as described by Shaw and colleagues [18], and spheres were counted 5 days after seeding.

### Patient ex vivo explants

**Fig. 1:**
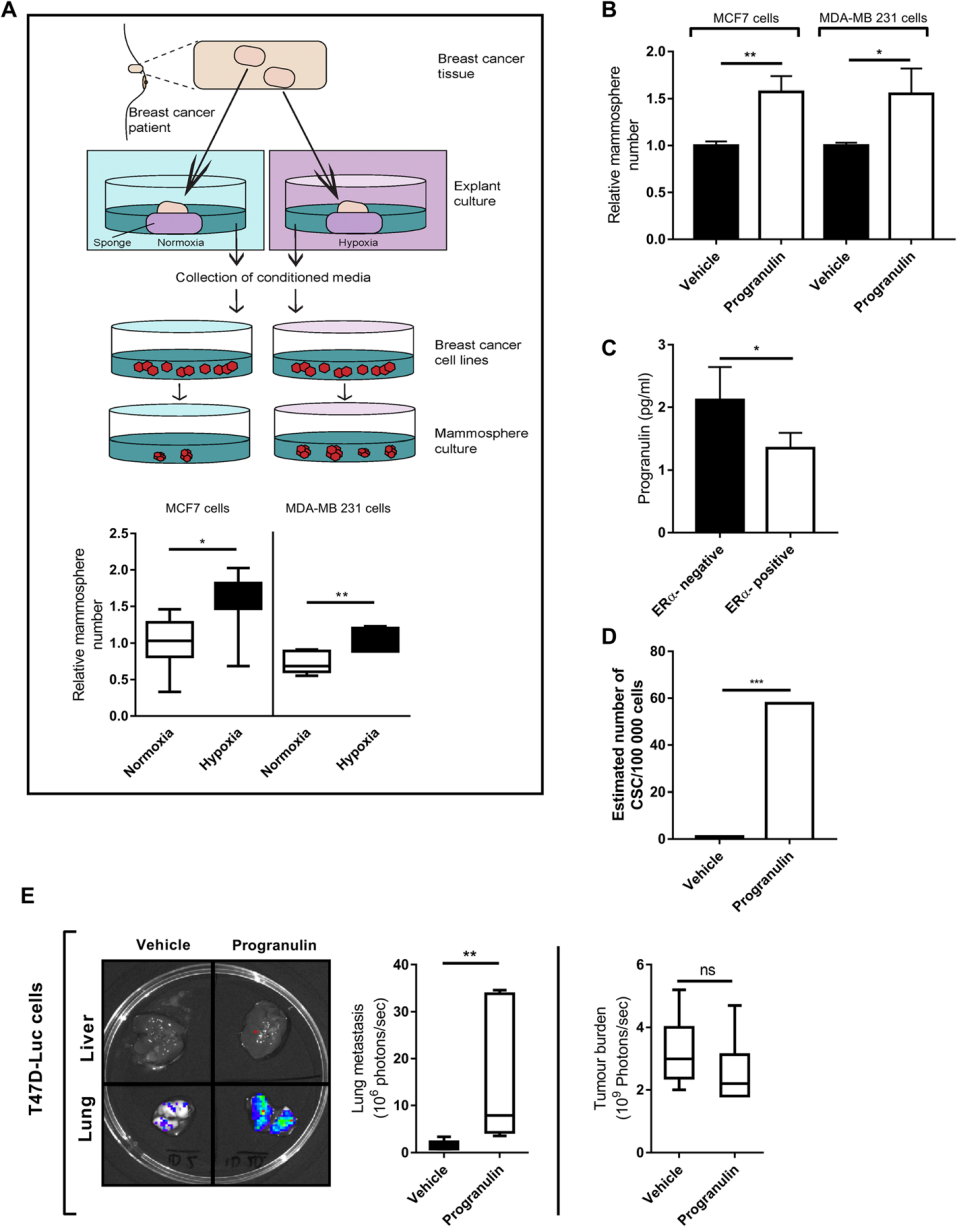
Identification of progranulin as a secreted component that influences breast cancer stem cell propagation. **a** Schematic of the experimental procedure involving ERα-positive primary tumour explants (*n* = 7) and mammosphere formation of breast cancer cell lines MCF7 and MDA-MB 231 in response to pre-treatment with conditioned media. Results are expressed as relative mammosphere formation (n = 7). *p < 0.05, **p < 0.01 and ***p < 0.001 as calculated by a Student’s *t* test. **b** ERα-positive MCF7 and ERα-negative MDA-MB 231 cell lines were treated with 1 μg/ml progranulin for 48 h and then assessed for mammosphere-forming capacity. Results are expressed as relative mammosphere numbers ± SD (*n* = 3). *p < 0.05, **p < 0.01 and as calculated by a Student’s *t* test. **c** Culture media collected from ERα-positive MCF7, T47D and ERα-negative MDA-MB 231 and MDA-MB 468 cultures where analysed for progranulin secretion using human progranulin ELISA (n = 3). *As calculated by a Student’s *t* test. **d** ERα-positive MCF7 cells were pre-treated with 1 μg/ml progranulin for 48 h and then injected into NOD SCID gamma mice in serial dilution format. Xenograft results were calculated at day 59 using extreme limiting dilution analysis (ELDA) software to determine the CSC frequency and significance. *p < 0.05, **p < 0.01 and ***p < 0.001. **e** T47D-luc xenografts where treated with either vehicle (PBS) or 8 μg of progranulin three times per week for 6 weeks by subcutaneous injection. Tumour burden and lung metastases luciferase measurements at the experimental endpoint are expressed as mean photons/second (*right, top and bottom* respectively) (*n* = 6). Mann-Whitney *U* test was used for statistics. **p < 0.01. *CSC* cancer stem cell, *ERα* estrogen receptor alpha

Breast cancer tissue was obtained with written informed consent through Sahlgrenska University Hospital, Gothenburg, Sweden. Five hundred micrometre-thick sections from breast cancer tumours collected from the pathologic department at Sahlgrenska using a vibrating blade microtome Leica, (Wetzlar, Germany) VT1000S and cultured in duplicate on a pre-soaked gelatin sponge (Johnson and Johnson, Pharmaceutical Research and New Research, Raritan, NJ, USA) in six-well plates containing DMEM-F12 (Lonza) (10% FCS/1% PenStrep) (illustrated in Fig. 1a [19, 20]) without covering the tissue. Tissues were cultured in either normoxic (21% O_2_) or hypoxic (1% O_2_) conditions at 37 °C for 48 h and then formalin-fixed and paraffin-embedded or preserved in RNAlater (Invitrogen). Conditioned culture media was taken from explants and centrifuged at 300 rcf for 5 min to remove cellular debris. This conditioned media was then used to either treat cell lines or analysed using Western blot.

### In vivo studies

Cells were injected subcutaneously into two sites of the flank of NOD SCID gamma mice (Taconic, Denmark) Ninety-day slow release estrogen pellets (0.72 mg, Innovative Research of America, Sarasota, FL, USA) were implanted subcutaneously 2 days before injection when using T47D only. Cells were suspended in a 1:1 mixture of matrigel (growth factor reduced) (BD Biosciences, San Jose, CA, USA) and mammocult media (Stemcell Technologies, Vancouver, BC, Canada). From tumour initiation studies cells were injected in a serial dilution in a 60% mixture of matrigel (growth factor reduced) and 40% complete media. For disease progression studies, MDA-MB 231 or T47D luciferase tagged cells were injected at a concentration of 0.2 × 10^6^ cells and 1 × 10^6^ cells, respectively, per injection site. The size of the tumours was determined by calliper measurement of the subcutaneous tumour mass two times per week. For sortilin inhibition studies, mice were given 5–10μg AF38469 (MedChem Express, Monmouth Junction, NJ, USA)/day/mouse in their drinking water. Water was changed weekly. Tumour volume was calculated according to the formula volume = (length × (width)^2^/2. The extreme limiting dilution analysis (ELDA) calculation was used to estimate stem cell frequency in treatment groups as described previously [21]. Assessments of metastasis were performed using the IVIS whole body imager (PerkinElmer, Waltham, MA, USA) based on luciferase expression from stably transfected cell lines.

### Transient transfections

MCF7 and T47D cells were transfected with 10 μM of siSORT1 (Origene, Rockville, MD, USA). Cells were seeded in T25 tissue culture flasks in PenStrep-free culture media. Cells were incubated at 37 °C 5% CO_2_ overnight. For each transfection, 500 μL of OptiMEM was mixed with 10 μL Lipo2000 (Solution A), and 500 μL OptiMEM was mixed with 10 μL siRNA (Solution B). Both solutions were incubated at room temperature for 5 min. Following incubation, both Solution A and Solution B were mixed together (transfection solution) and incubated for 20 min at room temperature. Cells were washed in PBS and transfection solution was added directly to the cells. Cells were then incubated for 4 h at 37 °C 5% CO_2_. After incubation, 4 mL of culture media was added to each T25 tissue culture flask. Cells were then incubated for 48 h in either normoxic (21% O_2_) or hypoxic (1% O_2_) conditions, or treated with 1 μg/mL progranulin (Nordic Biosite) for indicated time periods.

### Lentivirus transduction

A SOX2 pluripotency response reporter construct containing a SOX2 response element upstream of a minimal CMV promotor driving copGFP and luciferase was purchased from BioCat GmbH, Heidelberg, Germany as pre-packaged lentiviral particles. For lentiviral infection, T47D cells were seeded into six-well plates and incubated with lentiviral particles at a multiplicity of infection (MOI) of 1.5 in the presence of 5 μL/mL protamine sulphate (Sigma-Aldrich, St. Louis, MO, USA). The lentivirus solution was replaced with normal media 24 h later. Cells were cultured for 3 days to await the development of GFP signal at which point they were passaged and selected using puromycin.

### Western blotting

Cells were lysed in lysis buffer containing 50 mM HEPES, 150 mM NaCl (4.38 g), 1 mM EDTA, 1% (*w*/*v*) CHAPS and Sigma protease inhibitor cocktail (Sigma-Aldrich). Subsequently, the cell lysates were either boiled or unheated in 2 × SDS-PAGE loading buffer for 10 min and then 25 μg of protein was separated on a 12% SDS-PAGE and transferred to Hybond-C Extra nitrocellulose membrane (GE Healthcare Life Sciences, Chicago, IL, USA). Note, for progranulin detection, non-reducing conditions were used as these conditions were optimal for the primary antibody. As no loading control antibody preformed optimally using non-reducing conditions, ponceau stain was used to account for normalisation. Primary antibodies included: anti-tubulin (Abcam, Cambridge, MA, USA, #ab78109), anti-progranulin (R&D Systems, #af2420), anti-sortilin (Abcam, #ab16640), and anti-HIF-1α (BD Biosciences, #610959), anti-snail (Cell Signal Technology, Danvers, MA, USA, #C15D3), Anti-slug (Cell Signal Technology, #C19G7), anti-β-tubulin (Abcam, #6046), anti-β-actin (Santa Cruz Biotechnology, Dallas, TX, USA, #sc-1616). Protein detection was carried out using ECL (GE Healthcare Life Sciences).

### Single-cell qPCR analysis

Single cell analyses were performed as earlier described [22]. The analyses of primary tumour samples were analysed according to the protocol described in Akrap et al. [23].

### FACS sorting

Samples were resuspended at ≤1 × 10^6^ in 100 μL sorting buffer (5% FBS/1%PenStrep in PBS w/o MgCl_2_ and CaCl_2_) and incubated with 10 μL of primary PE conjugated CD24 antibody (#IM1428U, Beckman Coulter, Brea, CA, USA) for 20 min at 4 °C. Following incubation the cells were washed with 1 mL PBS and centrifuged at 300 rcf for 2 min. Cells were resuspended in 500 μL of sorting buffer and passed through a 100 μM sieve. Cells were sorted using the Becton Dickinson FACS ARIA II. Unlabelled cells were used to allow for the auto-fluorescence of the test cells. For single cell sorting, viable single cells were sorted into a Costar 3903 96-well plate (Thermo Fisher Scientific) for single cell analysis as earlier detailed [22] and for progranulin treatment of CD24^high^ and CD24^low^ cells, populations were sorted and separated in a 24-well plate for culture.

### Operetta high-content imaging system

0.008 × 10^6^ T47D Sox2-GFP reporter cells were plated per well in a CellGrade 96-well plate (BRANDplates) and allowed to attach for 24 h. Cells were treated with vehicle (PBS) or 1 μg/mL progranulin (Nordic Biosite) immediately prior to being imaged with an Operetta high-content imaging system (PerkinElmer) at ×20 magnification every third hour for 72 h at 37 °C 5% CO_2_. Images were analysed using Harmony software (Lausanne, Switzerland).

### ELISA

Culture media collected from ERα-positive MCF7, T47D and ERα-negative MDA-MB 231 and MDA-MB 468 cultures where analysed for progranulin secretion using human progranulin enzyme-linked immunosorbent assay (ELISA) (R&D Systems). The ELISA was performed accordingly to the protocol provided by the manufacture.

### Immunofluorescence

Cells were seeded onto chamber slides (Merck Millipore Ltd., Watford, UK) at 3 × 10^4^ cells/chamber for 48 h where after fixing with 4% paraformaldehyde (Histolab Products AB, Västra Frölunda, Sweden) in PBS for 5 min at room temperature (RT). Fixed cells were washed twice with PBS. After washing with PBS, the slides were blocked with 1% BSA for 45 min before incubated with CD24 (Invitrogen, #MA5–11833) diluted in 1% BSA/PBS overnight. Cells were washed three times with PBS, followed by incubation with Alexa Fluor 568 conjugated anti-mouse (Invitrogen #A11004) dissolved in 1% goat serum/1% BSA for 1 h at RT. After washing three times with PBS, the coverslips were stained with DAPI (Sigma-Aldrich) for 3 min. Stained cells were washed twice with PBS and once with water before cells were mounted on cover glass slides with prolong diamond anti-fade mountant media (Life Technologies, Carlsbad, CA, USA). Cells were examined under a Zeiss Axio Vert.A1 microscope (Carl Zeiss, Oberkochen, Germany) at ×5 magnification.

### Immunohistochemistry

Tissue sections were fixed with 4% phosphate-buffered formaldehyde, embedded in paraffin and cut into 4.5-μm-thick sections. Immunohistochemistry was performed using Dako Autostainer LINK 48 using Envision FLEX+ detection system (Dako, Glostrup, Denmark). Briefly, deparaffinized sections were subjected to antigen retrieval by high-pressure cooking and DIVA antigen retrieval pH 6.2, followed by blocking with 3% hydrogen peroxide and incubation with primary antibody against anti-Ki67 (monoclonal mouse anti-human Ki-67 antigen clone MIB-1. M7240 (Dako) and anti-sortilin antibody (polyclonal rabbit anti-sortilin, #AB16640, Abcam) at RT for 1 h. For signal amplification EnVision™ FLEX+ rabbit linker, SM805, (Dako) and EnVision™ FLEX+ mouse linker SM804 (Dako) was used, respectively. Further EnVision FLEX/HRP visualization reagent EnVision™ FLEX/HRP secondary antibody-coated polymer peroxidase complexes (#SM802, Dako), followed by DAB substrate/chromogen (Dako) was used. Slides were counterstained with Dako hematoxylin. Stained sections were scanned by Leica SCN400 scanner at 20× and evaluated by the automated image analysis Definiens Developer XD tissue studio program.

### RNA extraction, cDNA synthesis and qPCR

Total RNA was isolated using the TRIZOL reagent (Invitrogen). To quantify gene expression levels, equal amounts of cDNA were synthesized using the Advantage RT-for-PCR kit (Clontech, Mountain View, CA, USA) and mixed with the Power SYBR Green PCR master mix (Applied Biosystems, Carlsbad, CA, USA) and 5 pmol of both forward and reverse primers for pre-amplification as explained by Ståhlberg et al. [22]. All quantitative polymerase chain reaction (qPCR) performed using SYBR Green was conducted at 65 °C for 2 min, 95 °C for 10 min, and then 45 cycles of 95 °C for 15 s and 60 °C for 1 min. Primers used are listed on Additional file 1: Table S1. The specificity of the reaction was verified by melt curve analysis. The threshold crossing value was noted for each transcript and normalized to the internal control. The relative quantitation of each mRNA was performed using the comparative Ct method. Experiments were performed using an ABI Prism 7900 System (Applied Biosystems), and data processing was performed using GenEx software (GenEx Technologies, Bethesda, MD, USA).

### Statistical analysis

Multivariate analysis was performed using Stata 10 data analysis software (Stata Corp. LP, College Station, TX, USA) and was carried out using Cox’s proportional hazard model, using the Breslow method for ties. Mann-Whitney *U* test was used for non-parametric test, Fischer’s exact test for two by two tables and two-tailed Student’s *t* test was applied for continuous variables.

## Results

### Identification of progranulin as a secreted component that influences cancer stem cell-like propagation in breast cancer

While studying how different growth conditions influenced the fraction of cancer stem cells in breast cancer using mammosphere-forming capacity as a surrogate marker [18], we discovered that cells seemed to communicate using secretory signalling. Transfer of conditioned media from ERα-positive cell lines cultured in a hypoxic environment increased the mammosphere-forming capacity in both ERα-negative and ERα-positive breast cancer cell lines compared to normoxic conditioned media (Additional file 1: Figure S1A). Similar findings were obtained using conditioned media from primary breast cancer explants, thereby validating the results using intact breast cancer tissue (Fig. 1a). In order to identify secreted molecules that mediated the hypoxia induced mammosphere increase, conditioned media from ERα-positive cell lines cultured in hypoxic environment was analysed using a cytokine array. We found that progranulin was identified as a potent cytokine, increasing the mammosphere-forming capacity in both ERα-positive and ERα-negative breast cancer as illustrated in Fig. 1b. Further, we did not observe any increased progranulin secretion in ERα-negative breast cancer during hypoxia but instead a higher normoxic secretion compared to ERα-positive breast cancer cell lines (Fig. 1c). In addition, we have observed an increase in protein expression of the transcription factors SNAIL (SNAI2) and SLUG (SNAI1) in progranulin-treated breast cancer cell lines (Additional file 2) suggesting that progranulin also increase epithelial-mesenchymal transition (EMT) features of breast cancer cells. Limiting dilution experiments using NOD SCID gamma mice also showed that progranulin pre-treatment of cancer cell lines for 48 h prior to injection increased the frequency of cancer stem cells (Fig. 1d and Additional file 1: Figure S1B). Collectively, these results suggest that progranulin indeed induces cancer stem cell propagation in different subtypes of breast cancer and that progranulin is secreted in both ERα-negative and positive breast cancers during different in vivo relevant conditions.

### Progranulin induces metastasis in breast cancer models

Next, we tested how progranulin influenced breast cancer growth and progression in vivo by repetitive injections of the protein in tumour-bearing mice using luciferase-tagged breast cancer cell line xenografts. Two weeks of progranulin treatment during xenograft growth did not significantly influence T47D (Fig. 1e right) or MDA-MB 231 (Additional file 1: Figure S2 right) xenograft tumour burden. Nevertheless, there was a significant increase of lung metastases in both ERα-positive (Fig. 1e left) and ERα-negative models (Additional file 1: Figure S2 left). These data suggest that progranulin affects tumour progression and a metastasising cellular subtype.

### The sortilin receptor is a prerequisite for progranulin-induced CSC-like propagation

**Fig. 2:**
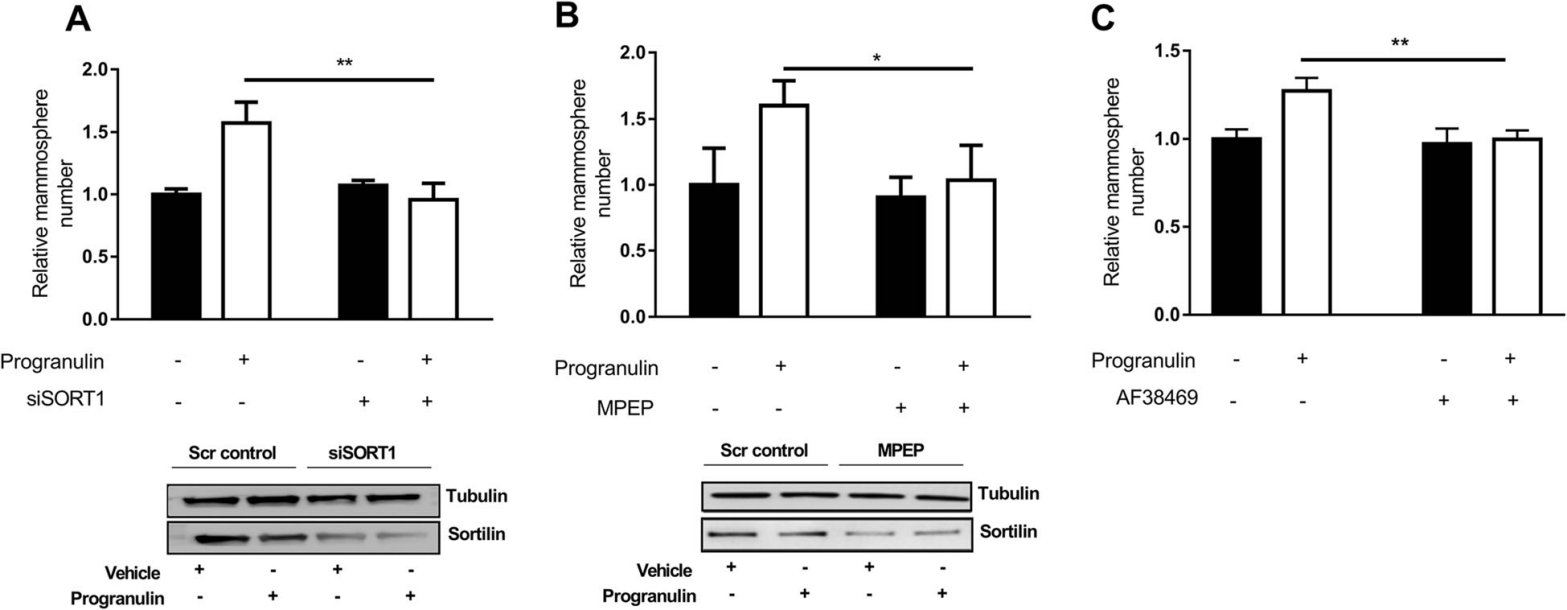
The receptor sortilin is a prerequisite for progranulin-induced CSC-like propagation. **a** MCF7 cells treated with siRNA against sortilin or scrambled control were pre-treated with either vehicle (PBS) or 1 μg/ml progranulin and analysed for mammosphere-forming capacity and protein expression (Western blot). Results are expressed as relative mammosphere number ± SD (*n* = 3). **p < 0.01 as calculated by a Student’s *t* test. Western blot analysis of sortilin expression confirmed sortilin knockdown. **b** MCF7 cells treated with either vehicle or the sortilin-degrading drug MPEP (10 μM). 3 h later cells were treated with either vehicle (PBS) or 1 μg/ml progranulin for 48 h and analysed for mammosphere-forming capacity. Results are expressed as relative mammosphere number ± SD (n = 3) (*top*). *p < 0.05 as calculated by a Student’s *t* test. Western blot analysis of sortilin expression confirmed sortilin degradation after MPEP (10 μM) treatment (*bottom*). **c** MCF7 cells treated with either vehicle (PBS) or 1 μg/ml progranulin with or without the small molecule AF38469 (10 μM) and analysed for mammosphere-forming capacity. Results are expressed as relative mammosphere number ± SD (*n* = 3). **p < 0.01 as calculated by Student’s *t* test, *MPEP* 1-[2-(2-tert-butyl-5-methylphenoxy)-ethyl]-3-methylpiperidine

In order to identify how progranulin induced cancer stem cell propagation we investigated the role for the receptor sortilin, previously identified as one of the main receptors for progranulin [24, 25]. Silencing of sortilin using siRNA-inhibited progranulin induced increase in mammosphere formation of both MCF7 (Fig. 2a) and MDA-MB 231 cells (Additional file 1: Figure S3A). Similar results were obtained when treating cells with the recently identified sortilin affecting compound 1-[2-(2-tert-butyl-5-methylphenoxy)-ethyl]-3-methylpiperidine termed MPEP [16] (Fig. 2b and Additional file 1: Figure S3B). The small orally available sortilin inhibitor AF38469 [26] was also tested and effectively inhibited the progranulin-mediated mammosphere-forming capacity of MCF7 cells (Fig. 2c) and MDA-MB 231 cells (Additional file 1: Figure S3C) without affecting proliferation and cell viability (Additional file 3). Taken together, these results demonstrate that progranulin-induced cancer stem cell-like propagation is dependent on sortilin expression and function.

### Progranulin and sortilin are mainly expressed in differentiated and proliferative cells in breast cancer

**Fig. 3:**
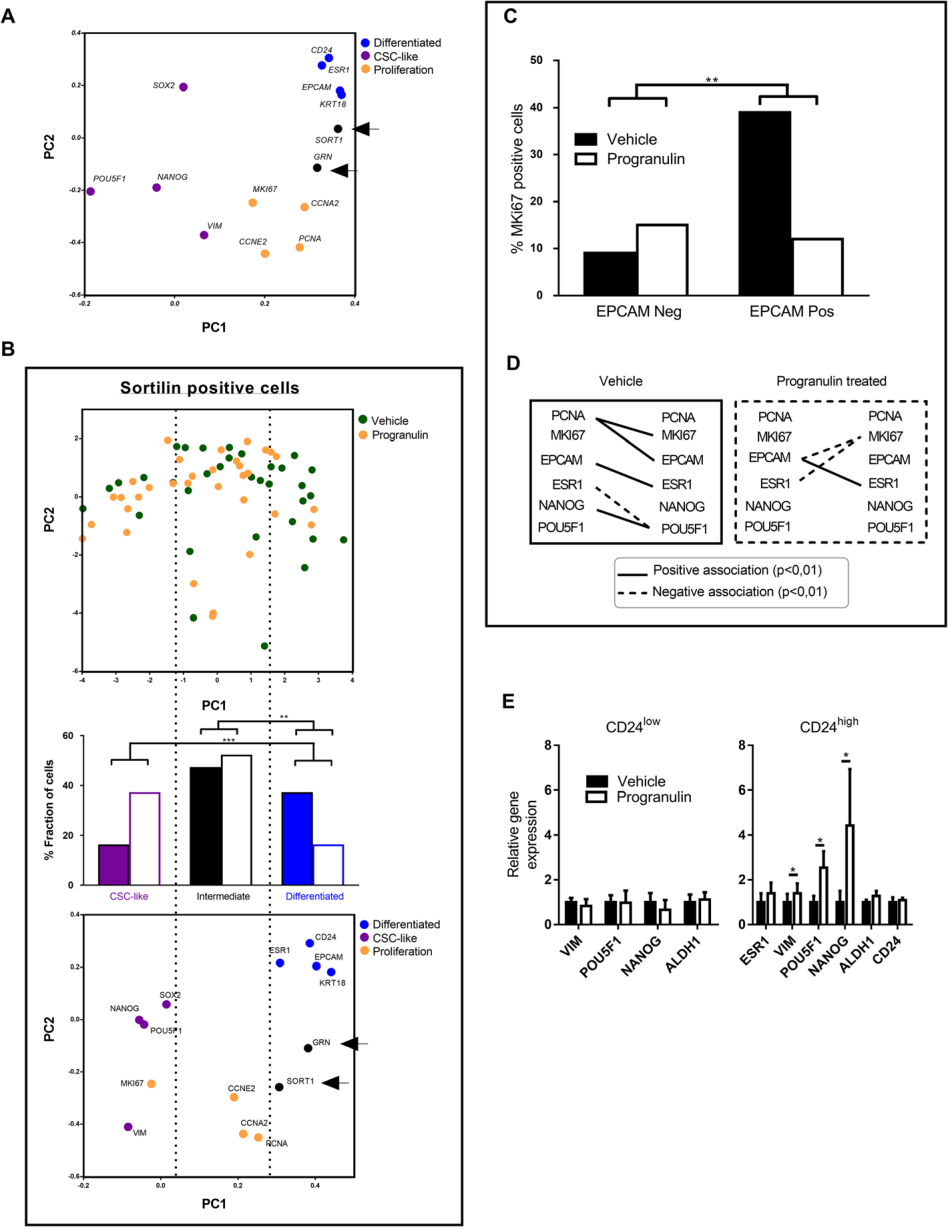
Single-cell analyses of breast cancer cells illustrating dedifferentiation of sortilin-positive cells after progranulin treatment. **a** PCA illustrating the separation of gene clusters based on transcriptional expression. *SORT1* and *GRN* clustered with differentiation (*blue*) and proliferation gene clusters (*yellow*) while CSC-like transcripts cluster separately (*purple*). **b** The analysis of the transcriptional associations of single-cell gene expression with each cell represented by a circle of vehicle- and progranulin-treated MCF7 cells only including *SORT1*-positive cells. Individual cells are indicated in the *top PCA score plot* and the fractions of cells in relation to differentiation and CSC-like properties are summarised in the *middle panel* whereas the PCA loading plot and clustering for PC1 and PC2 are shown in the *bottom panel*. **p < 0.01 and ***p < 0.001 as calculated by Fisher’s exact test. **c** *MKI67* (% positive cells) expression assessment in relation to the expression of *EPCAM* in *SORT1*-positive single cells treated with vehicle control or progranulin. **p < 0.01 as calculated by Fisher’s exact test. **d** Correlation analyses between single-cell transcripts in vehicle- or progranulin-treated MCF7 cells. *Black line* represents a significant (*p* < 0.01) positive association and a *dotted line* indicates a significant (*p* < 0.01) negative association. Correlation co-efficient is calculated by Spearman’s correlation. **e** CD24^low^ (*left*) and CD24^high^ (*right*) cells were treated with vehicle or progranulin and bulk expression of selected transcripts were measured by qPCR and represented as relative gene expression compared to vehicle treated cells (n = 3). *p < 0.05 as calculated by a Student’s *t* test. *CSC* cancer stem cell

To define potential cellular subpopulations responsible for progranulin secretion, as well as the reacting sortilin-expressing population, we performed single-cell gene expression profiling using qPCR and used signature genes for differentiation, CSC-like/EMT properties, progranulin signalling (*GRN, SORT1*) and proliferation as earlier established [23]. Data showed that approximately 78% of the analysed MCF7 cells (n_total_ = 60) expressed *GRN* and that the gene expression clustered together with proliferation and differentiation markers as illustrated by principle component analysis (PCA) (Fig. 3a). In support of the gene expression clustering in the PCA, *GRN* expression was significantly associated with that of *PCNA, CCNA2, EPCAM, KRT18, CD24* and *ESR1*, as well as inversely associated with the CSC- linked transcript *POU5F1* (Additional file 1: Figure S4 left). *SORT1* was expressed in 57% of all MCF7 cells and its expression correlated with that of *GRN* (Fig. 3a). Similar to *GRN, SORT1* expression correlated on a single-cell level with differentiation and proliferation markers and was inversely associated with the CSC-marker *POUF51* (Additional file 1: Figure S4 right). These data suggest that a highly differentiated and proliferative subpopulation of MCF7 cells expressed both the hypoxia-induced secreted molecule progranulin as well as the responding receptor sortilin, implying an autocrine signalling pathway.

### Progranulin induces cancer stem cell propagation by dedifferentiation and increased proliferation of the cancer stem cell pool

Interestingly, when restricting the analyses to the *SORT1*-positive subpopulation and using PCA to illustrate the alterations in single-cell gene expression, progranulin treatment caused a gradual shift from highly differentiated cell types to more CSC-like cells (Fig. 3b). Moreover, single-cell analyses demonstrated that *EPCAM* and *SORT1* double-positive differentiated cells clearly became less proliferative in response to progranulin treatment, while there was no significant difference in the *EPCAM*-negative subpopulation of cells (Fig. 3c). Further, progranulin caused major changes in the properties of subgroups of cancer cells as illustrated by association plots (Fig. 3d). These data imply that there was a general increase of CSC-like proliferative cells in the *SORT1*-positive subpopulation after progranulin treatment whereas proliferation of remaining *SORT1*-positive cells and differentiated cells decreased. Moreover, progranulin treatment of CD24^low^- and CD24^high^-sorted MCF7 cells did not induce any transcriptional changes in the CD24^low^ subpopulation, but clearly affected the CD24^high^ subpopulation with significant increases in CSC-associated gene expressions (Fig. 3e), suggesting that differentiated CD24^high^ cells respond to progranulin and transform into a more CSC-like population.

**Fig. 4:**
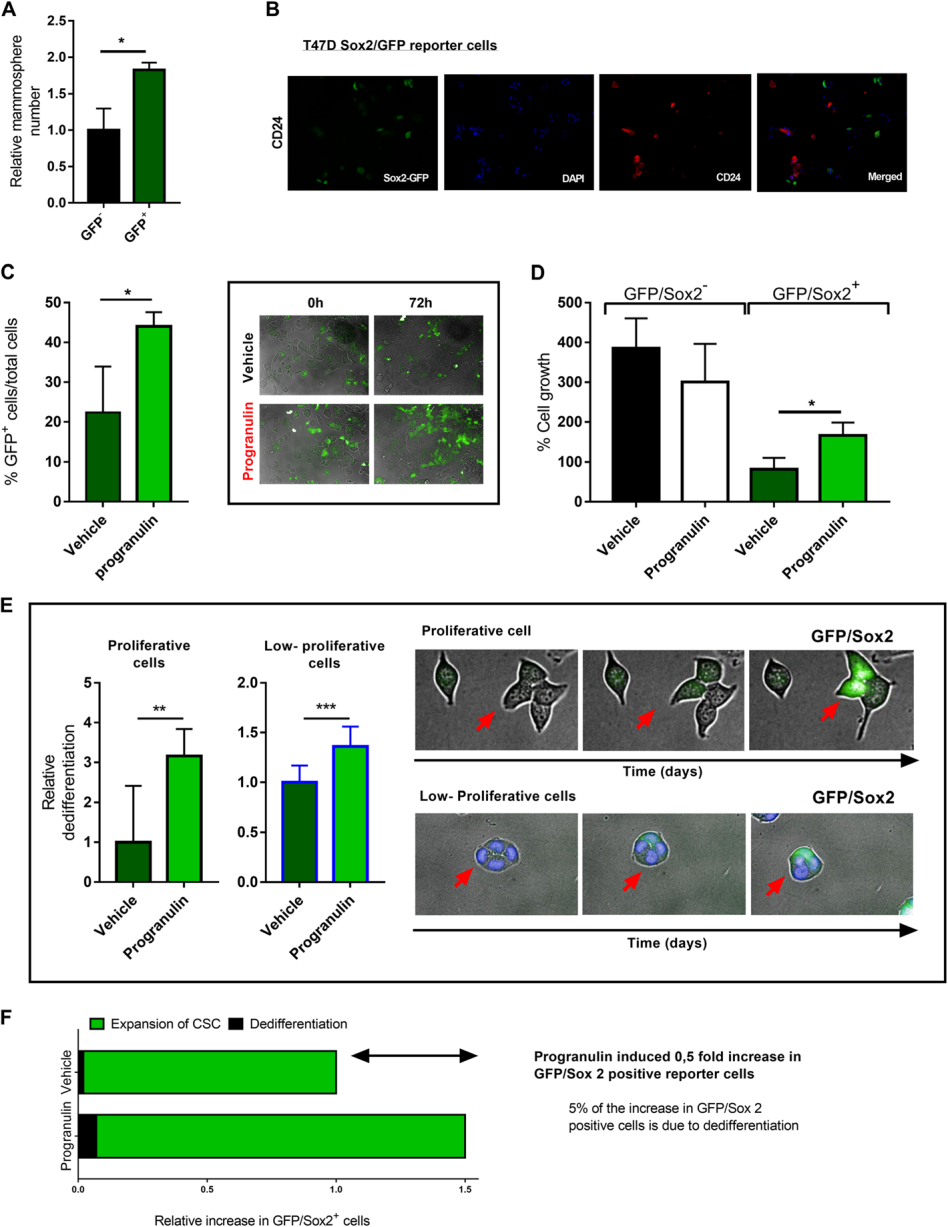
Progranulin induces cancer stem cell propagation by dedifferentiation and increased proliferation of cancer stem cells. **a** FACS sorted GFP-positive and GFP-negative T47D GFP-Sox2 promotor reporter cells were analysed for mammosphere-forming capacity. Results are expressed as relative mammosphere number ± SD (*n* = 2). *p < 0.05 as calculated by a Student’s *t* test. **b** Immunofluorescence staining of CD24 (*red*), GFP (*green*), DAPI (*blue*) expression of GFP-Sox2 reporter cells. **c** (*Left*) Frequencies of GFP-positive cells were analysed using Operetta high-content imaging system. Frequencies of GFP-positive cells among all cells at 72 h were calculated after vehicle (PBS) (*dark green*) or 1 μg progranulin (*light green*) treatment (n = 3). *p < 0.05 as calculated by a Student’s *t* test. **c** Images illustrating the GFP expression in cells at starting time and after 72 h of progranulin treatment. **d** Percentage cell growth of GFP-Sox2 reporter cells at 72 h of treatment (n = 3). *p < 0.05 as calculated by a Student’s *t* test. **e** (*Left bar plots*) Nine fields in three 96-well (three fields each well) with unstained GFP-Sox2 reporter cells or nuclear-stained cells with NucBlue (*right*) where analysed over time to observe changes in GFP expression. Cells were considered as dedifferentiated cells if they turned GFP positive during 72 h of treatment. **p < 0.01 and ***p < 0.001 as calculated by a Student’s *t* test. **e** (*Right images*) Example of differentiated cells with both unstained (proliferative) and NucBlue-stained cells (low-proliferative). **f** Illustrative summary of the expansion process of T47D GFP-Sox2-positive reporter cells after progranulin treatment. *CSC* cancer stem cell

In order to further address the role of dedifferentiation of cancer cells after progranulin treatment, we used a GFP-Sox2 response reporter cell line (BioCat GmbH) and monitored the Sox2-expressing cells during progranulin treatment using the Operetta high-content imaging system. Initially, we tested the mammosphere-forming ability of the reporter cells and as expected, GFP-positive cells had a significantly increased mammosphere-forming ability compared to GFP-negative cells (Fig. 4a). Immunofluorescence analysis further demonstrated that GFP-Sox2-positive cells did not co-express CD24 (Fig. 4b), indicating that GFP-Sox2-positive cells exhibited a more CSC-like phenotype. Further, treating the GFP-Sox2 reporter cells with progranulin for 72 h did not change total cell proliferation and absolute cell numbers compared to vehicle control (Fig. 4d); however, there was a marked increased ratio of GFP-positive cells compared to the vehicle control (Fig. 4c). When examining the expansion of GFP-negative versus GFP-positive cells 72 h after progranulin treatment there was a clear expansion of GFP-positive cells but no significant change in GFP-negative cells (Fig. 4d). Interestingly, when adding nuclear staining to the culture for accurate automated image visualisation, we observed a marked decreased proliferation of cells in all culture conditions (Additional file 4). We also observed a higher occurrence of GFP-negative cells turning GFP-positive compare to growth conditions with nuclear staining (Fig. 4e), suggesting that suppressed proliferation facilitates dedifferentiation of breast cancer. Importantly, progranulin-treated samples demonstrated a marked increase in GFP-negative cells turning GFP-positive under both conditions (Fig. 4e). Single-cell data and image analyses of reporter cells collectively support that progranulin induces true dedifferentiation of breast cancer cells as well as an increased proliferation of the cancer stem cell pool as summarised in Fig. 4f.

### The orally bioavailable small molecule AF38469 inhibits progranulin domain A-induced breast cancer metastases and local infiltrative growth in vivo

**Fig. 5:**
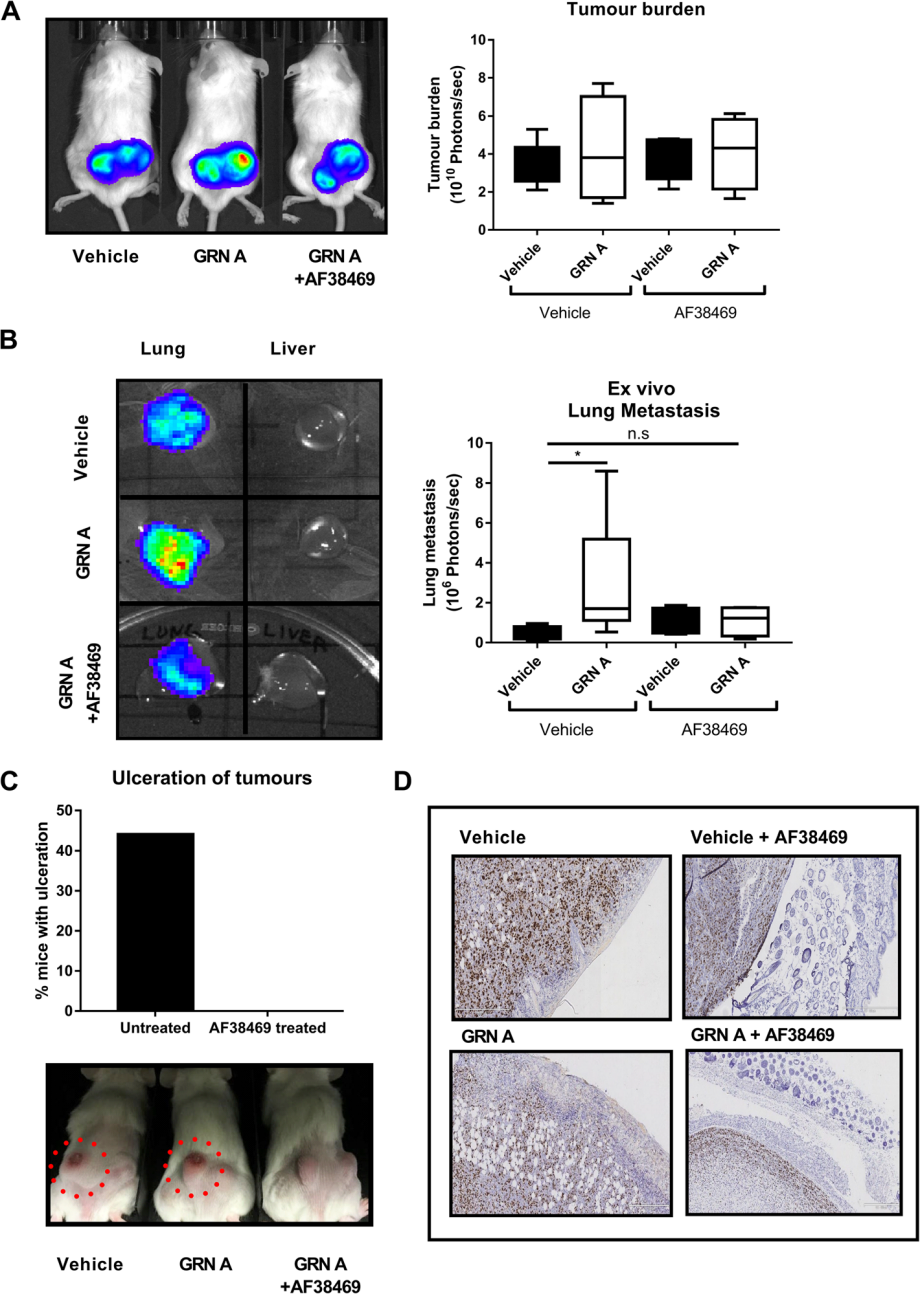
AF38469 inhibits progranulin domain A-induced breast cancer metastases and local infiltrative growth in vivo. MDA-MB 231-luc xenografts were injected with either vehicle (PBS) or 8 μg of GRN A three times per week for 6 weeks by subcutaneous injection. Mice were separated into groups (vehicle (*n* = 5), GRN A (n = 5), vehicle + AF38469 (*n* = 4) and GRN A + AF38469 (n = 5)). The sortilin inhibitor AF38469 or vehicle control was administered in drinking water at concentrations of 5 μg/mouse/day (n = 2) and 10 μg/mouse/day (n = 3). 5**–**10 μg AF38469 groups were pooled (total n = 5). **a** Tumour burden and (**b**) lung metastases luciferase measurements at the experimental endpoint are expressed as mean photons/second (*top and bottom*, respectively). Mann-Whitney *U* test where used for statistics. *p < 0.05. **c** (*Bottom*) Macroscopic visualisation of ulcerating tumours in AF38469-treated or vehicle-treated mice. (*Top*) Fraction of mice with tumour ulceration of mice injected with 8 μg of GRN A or GRN A in combination with AF38469 (*n* = 9). **d** Immunohistochemical analyses of the GRN A- and AF38469-treated groups of MDA-MB 231 luc xenografts with skin was performed using Ki67 and haematoxylin staining. Scale bar represents 5 × 250 μm. *GRN* granulin

Next, we verified the ability of the orally bioavailable small molecule AF38469 [26] to inhibit progranulin-induced disease progression in vivo by delivering AF38469 to the mice via their drinking water during tumour growth. Since we observed a marked inhibition of the progranulin effect in vitro using a protease inhibitor (protease inhibitor secretory leukocyte protease inhibitor - SPLI) blocking cleavage of full-length progranulin to small granulins (Additional file 1: Figure S5A) and that GRN A had a more pronounced effect in vivo compared to progranulin, (Additional file 1: Figure S2 (progranulin) compared to Fig. 5b (GRN A)) granulins are most likely important mediators of the progranulin effect. Therefore, in MDA-MB 231-luc xenografted mice experiments, mice were given AF38469 in their drinking water in parallel to repetitive injections of the active progranulin domain GRN A for 3 weeks. The results showed that GRN A did not change the tumour burden of the xenograft (Fig. 5a) but caused a significant increase in lung metastasis (Fig. 5b). Importantly, mice treated with AF38469 in conjunction with GRN A injections did not show any change in xenograft burden (Fig. 5a) compared to control but had a clear decreased formation of lung metastasis compared to mice not receiving the sortilin inhibitor (Fig. 5b and Additional file 1: Figure S5B). Further, immunohistochemical staining of the xenografts showed a decreased expression of sortilin after GRN A injections as well as in combination with AF38469 treatment (Additional file 1: Figure S5C), demonstrating that GRN A induces degradation of SORT1 similar to what has been shown with full-length progranulin [27]. Moreover, MDA-MB 231-luc xenografts grew highly infiltrative and produced ulcerations and skin infiltration in a large fraction of the control and GRN A-treated animals (Fig. 5c bottom images and Fig. 5d IHC images). Strikingly, all AF38469-treated mice lacked skin ulcerations, suggesting that sortilin inhibition blocked the local infiltrative behaviours of the xenograft (Fig. 5c). Altogether these data demonstrate that an active domain of progranulin (GRN A) affects tumour-progressing properties of breast cancer favouring CSC and EMT features, producing a metastasising and infiltrative cellular subtype, which can be inhibited by a sortilin inhibitor.

### SORT1 and GRN associations during primary human breast cancer progression in vivo

**Fig. 6:**
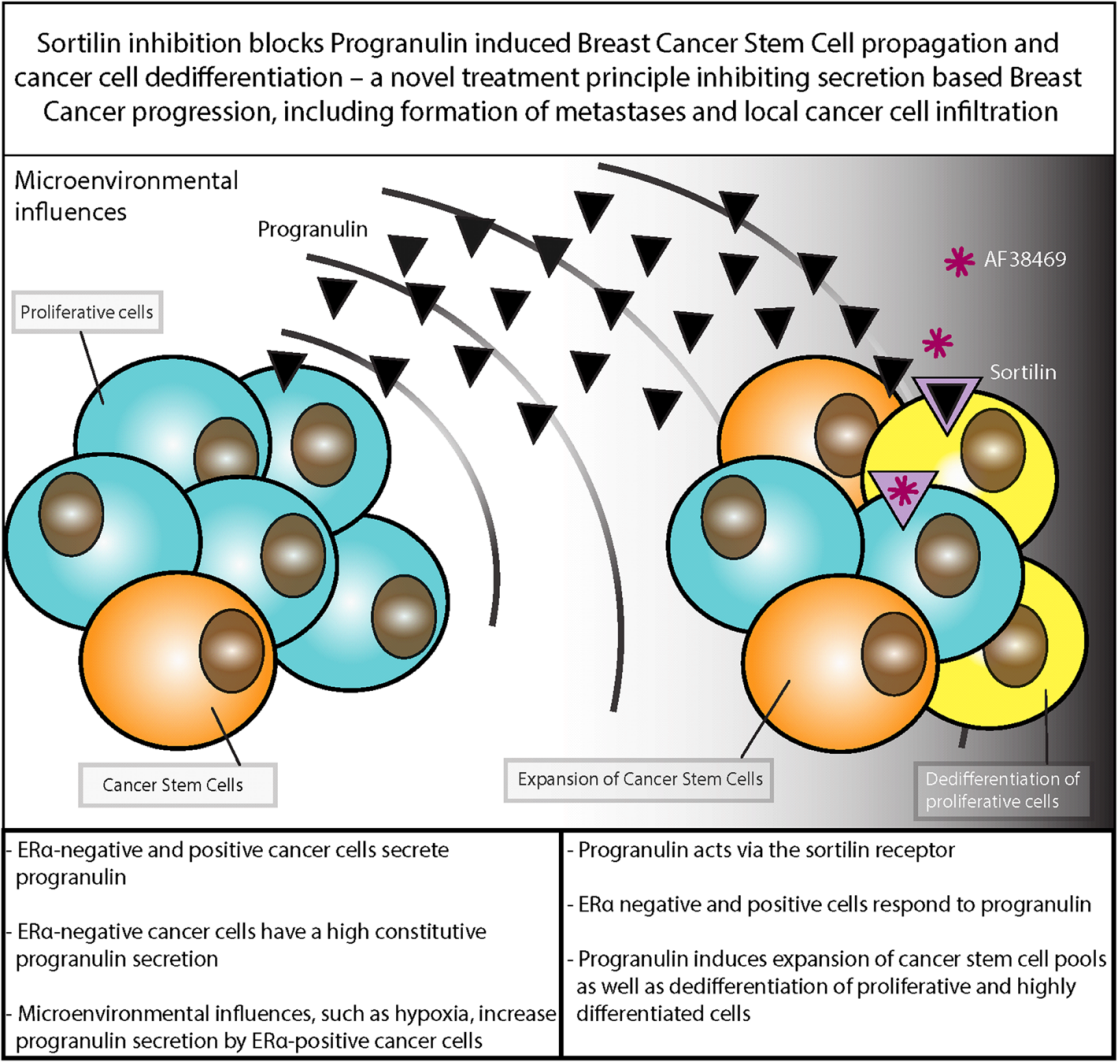
Summary of progranulin secretion and sortilin activation leading to breast cancer stem cell propagation. *CSC* cancer stem cell,

As summarised in Fig. 6, the presented data collectively supports that ERα-negative and ERα-positive breast cancer secrete progranulin under various in vivo-relevant growth conditions. Progranulin has the potential to act on sortilin-expressing differentiated cells and initiating tumour progression, partially via dedifferentiation, but also by increasing the proliferation of the cancer stem cell pool. In xenograft model systems, this change in proportions between subpopulations of cancer cells was paralleled by an increased metastasising potential. By inhibiting sortilin using the small molecule AF38469, the cancer-progressing and cancer stem cell-promoting features of progranulin in vivo could be blocked, reducing lung cancer metastases as well as completely preventing local skin infiltration of the xenograft models.

## Discussion

In recent years, it has been evident that the tumour microenvironment can influence biological processes in cancer, leading to aggressive phenotypes [28, 29]. Both stromal features and hypoxia have been highlighted as main contributors to microenvironmental cellular pressure [30–33]. A potential important mediator of these effects is secretion from various cells within the tumour, affecting nearby and more distant cancer cell populations, potentially spreading cancer-progressing properties. One area of major clinical and biological interest is the presence and regulation of CSC populations. Previous research has illustrated the importance for CSCs in tumour repopulation after conventional cancer therapy [34, 35]. High fractions of CSCs in various cancer types have also been linked to tumour aggressiveness, and it is clear that CSC properties are important in mediating clinically aggressive behaviours as well as treatment failures [36–38]. In light of these critical CSC functions, we present comprehensive data that define how secretion of progranulin can induce widespread CSC-promoting signalling in breast cancer, having a potential to influence future cancer therapy design.

The fact that ERα-positive breast cancer secreted elevated levels of progranulin under hypoxic stress whereas ERα-negative breast cancer constantly secreted high levels of progranulin, suggests that subtypes of breast cancers may have diverse responsiveness towards microenvironmental fluctuations and also different mechanisms for progranulin secretion. Nevertheless, the response to progranulin was similar in different subtypes of breast cancer with a clear CSC-propagating effect in all tested cell lines. The constant elevated progranulin secretion from ERα-negative breast cancer cells is in line with a reported high progranulin expression in ERα-negative breast cancer compared with other subtypes of the disease [13, 39]. Interestingly, ERα-negative breast cancer have high basal secretion of the cytokines interleukin (IL)-8 and IL-6, which have been linked to the pathophysiology of cancer, suggesting that ERα-negative breast cancer secretes cancerogenic, CSC- and growth-promoting cytokines [40–42] including progranulin. Our data further support that progranulin has the potential to function as an autocrine factor, but will probably also act in a paracrine fashion influencing nearby tumour cells. However, since subcutaneously injected progranulin showed pronounced effects on distant xenografts in mice, it suggests that progranulin may well have systemic effects potentially influencing a wide range of biological events.

In order to investigate how progranulin increased the fraction of CSC-like cells, we used different methods based on single-cell analyses. By exclusive analyses of the *SORT1*-positive subpopulation, using single-cell gene expression profiling, we could show an increase of CSC-like expression patterns in *SORT1*-positive cells during progranulin treatment. These changes were further paralleled by a pronounced proliferative decrease during progranulin treatment in *SORT1*-positive and differentiated cells, whereas non-differentiated and CSC-like cells showed a preserved proliferation. Similar results were obtained when using a Sox-2 reporter cell line, which showed an increased expansion of Sox-2-positive CSC-like cells after progranulin treatment. Importantly, we could follow Sox-2 reporter cells by time-lapse experiments where Sox2- GFP-negative cells converted into GFP-positive cells, strongly indicating a dedifferentiation process. Interestingly, this process was more pronounced under low proliferative conditions, which is in line with earlier data linking proliferation control with cancer stem cell features [43]. These results also highlight that dedifferentiation is an event that needs to be taken into account when it comes to in vivo growing cancer cells, as they generally have lower proliferation compared to standard cell culture model systems. Previously, reports have shown that the tumour microenvironment indeed provides stimuli for dedifferentiation processes [44, 45]. Chaffer and colleagues have demonstrated that breast cancer cells dedifferentiated into CSC due to transforming growth factor bets (TGFβ) microenvironmental signalling [44]. Another study suggests that colorectal stroma secrete growth factors that can induce stem cell features of differentiated colon cancer cells [45]. Further, the inflammatory cytokine tumour necrosis factor alpha (TNFα) secreted by cells in the microenvironment has been shown to induce dedifferentiation in melanoma tumours [46]. In summary, our results suggest that progranulin increases the cancer stem cell pool by maintaining and increasing the proliferation of CSC-like cells as well as inducing dedifferentiation.

The fact that we could show that progranulin mediates sortilin activation resulting in CSC propagation suggests that sortilin is a key molecule that genuinely affects tumour progression and metastatic properties. Further studies are needed in order to define the exact role for sortilin in this context, but our results clearly support an important role for sortilin in CSC propagation. A recent publication further supports the finding that sortilin is linked to breast cancer progression and disease recurrence in advanced disease [17]. The identification and clarification of the progranulin-sortilin cellular communication system linked to cancer-progressing properties, opens up a potential to block this tumour-promoting interplay offering a unique cancer treatment principle based on selective targeting of a partially microenvironmentally induced communication system.

In this study, we could demonstrate that the sortilin inhibitor AF38469, developed to treat frontal temporal dementia, also had a pronounced effect on progranulin-induced CSC propagation both in vitro and in vivo*.* AF38469 has previously been shown to bind as strongly as neurotensin to sortilin [26]. Here, we report that AF38469 treatment caused inhibition of progranulin and the progranulin domain A (GRN A) induced CSC propagation, both in vitro and in vivo, and metastasis in mice. Interestingly, we also observed that AF38469 treatment offered a complete protection against cancer cell infiltration from the xenograft into the skin. This was observed both macroscopically, by lack of ulcerations above the xenograft sites, as well as by intact skin after microscopical inspection. GRN A has previously been shown to play a critical role in epithelial homeostasis, tumorigenesis, and in reproductive, immunological, and neuronal functions [47, 48], demonstrating that this cleaved progranulin domain indeed possesses potent physiological activity. As to the best of our knowledge, AF38469 has not previously been demonstrated in relation to blocking progranulin stimulation of breast cancer cells and is an interesting bioavailable drug that can be further developed into breast cancer treatment protocols. Both anti-progranulin and anti-sortilin treatments should nevertheless be explored in more detail, as well as other potential progranulin receptors like the tumour necrosis factor receptor (TNFR) [49] and the newly identified progranulin receptor ephrin type-A receptor 2 (EPHA2) [50], in order to optimally target this communication system.

## Conclusions

In this study, we have identified that both ERα-negative and ERα-positive cancer cells secrete and respond to progranulin. Progranulin induces expansion of breast cancer stem cells as well as dedifferentiation of proliferative and highly differentiated cells via the sortilin receptor. Sortilin inhibition blocks progranulin-induced breast cancer stem cell propagation in vivo, which include protection from metastases and local cancer cell infiltration and ulceration of tumours.

## Additional files

Additional file 1: **Figure S1**. Progranulin is identified as a major contributor to hypoxic induction of mammosphere formation in breast cancer cell lines. **Figure S2**. Progranulin induces breast cancer metastases in vivo*.* **Figure S3**. The receptor sortilin is a prerequisite for progranulin-induced CSC-like propagation. **Figure S4**. (*Left*) *GRN* expression or (*right*) *SORT1* expression in MCF7 cells with selected functional transcripts. Correlation co-efficient is calculated by Spearman’s correlation and *p* values are indicated in the figure. **Figure S5**. The orally bioavailable small molecule AF38469 inhibits GRN domain A-induced CSC-like propagation and disease metastasis in vivo*.* **Table S1**. Sequence list for primers used during qPCR analysis. (DOCX 24130 kb)

Additional file 2: Increase in protein expression of the transcription factors SNAIL (SNAI2) and SLUG (SNAI1) in progranulin-treated MDA-MB 231 breast cancer cells. MDA-MB 231 cells treated with either vehicle (PBS) or 1 μg/ml progranulin (×) and analysed for protein expression (Western blot). *Left box* illustrates protein ladder and targeted proteins at different exposure times. (TIFF 1453 kb)

Additional file 3: The small orally available sortilin inhibitor AF38469 do not impact cell viability. Relative proliferation of MCF7 and MDA-MB 231 cells 48 h after treatment with vehicle (PBS/DMSO), AF38469 (3 μg/ml), progranulin (PGRN) (1 μg/ml) or AF38469 (3 μg/ml) and PGRN (1 μg/ml) by Alamar Blue assay. (TIFF 134 kb)

Additional file 4: Decreased proliferation of nuclear stained GFP-Sox2 reporter cells at 72 h of treatment. Percentage total cell growth of GFP-Sox2 reporter cells at 72 h of treatment with PBS or progranulin (PGRN) (1 μg/ml). *Left bar plots* demonstrate nuclear-stained cells with NucBlue and *right bar plot* demonstrate unstained GFP-Sox2 reporter cells. (TIFF 110 kb)

## Abbreviations

CSC: Cancer stem cells
DMEM: Dulbecco modified Eagle’s medium
ELDA: Extreme limiting dilution analysis
ELISA: Enzyme-linked immunosorbent assay
EMT: Epithelial–mesenchymal transition
EPHA2: Ephrin type-A receptor 2
ERα: Estrogen receptor alpha
FCS: Fetal calf serum
GRN: Granulin
MMP12: Neutrophil elastase and matrix metallopeptidase 12
MPEP: 1-[2-(2-tert-butyl-5-methylphenoxy)-ethyl]-3-methylpiperidine
PCA: Principle component analysis
PCDGF: Prostate cancer cell-derived growth factor
qPCR: Quantitative polymerase chain reaction
RPMI: Roswell Park Memorial Institute
SPLI: Protease inhibitor secretory leukocyte protease inhibitor
TNFR: Tumour necrosis factor receptor
