# Supplementary material for: Sortilin inhibition limits secretion-induced progranulin-dependent breast cancer progression and cancer stem cell expansion
Source: Breast Cancer Res. 2018 Nov 20;20:137. doi: 10.1186/s13058-018-1060-5 (PMC6245804; doi:10.1186/s13058-018-1060-5)
Supplement: Supplementary file 1 — Figure S1. Progranulin is identified as a major contributor to hypoxic induction of mammosphere formation in breast cancer cell lines. Figure S2. Progranulin induces breast cancer metastases in vivo. Figure S3. The receptor sortilin is a prerequisite for progranulin-induced CSC-like propagation. Figure S4. (Left) GRN expression or (right) SORT1 expression in MCF7 cells with selected functional transcripts. Correlation co-efficient is calculated by Spearman’s correlation and p values are indicated in the figure. Figure S5. The orally bioavailable small molecule AF38469 inhibits GRN domain A-induced CSC-like propagation and disease metastasis in vivo. Table S1. Sequence list for primers used during qPCR analysis. (DOCX 24130 kb) [file 13058_2018_1060_MOESM1_ESM.docx]

**Supplementary figures and figure legends**


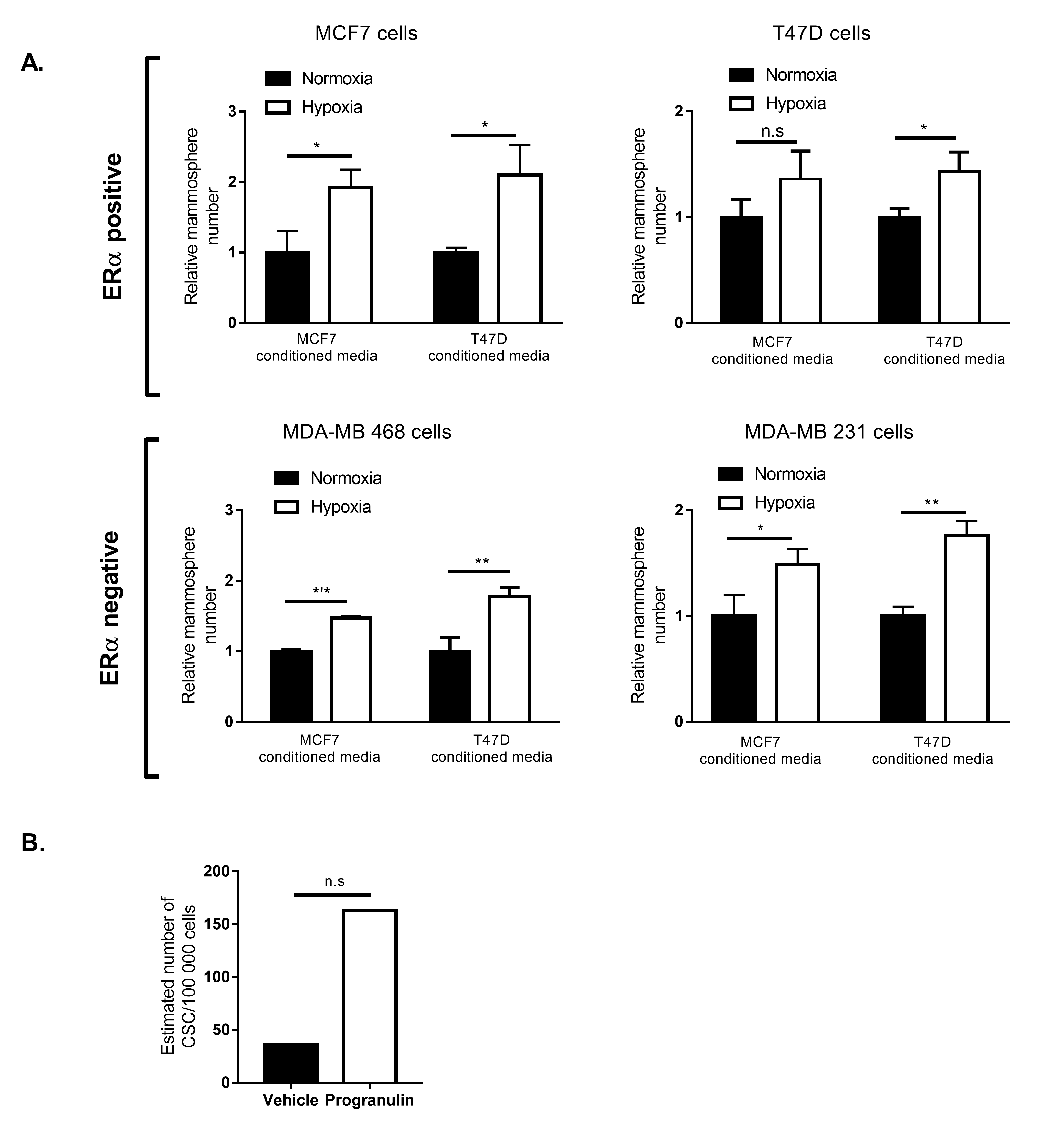


**Figure S1. Progranulin is identified as a major contributor to hypoxic induction of mammosphere formation in breast cancer cell lines.**

**(A)** ERα-positive T47D, MCF7 and ERα-negative MDA-MB 231 and MDA-MB 468 cells where pre-treated with conditioned media from ERα-positive MCF7 and T47D cells and then assessed for mammosphere forming capacity. Results are expressed as relative mammosphere formation ± SD of three independent experiments. * indicates p<0.05, ** p<0.01 and *** *p*<0.001 as calculated by a student’s t-test. (**B**) ) ERα-negative MDA-MB 231 cells were pre-treated with 1µg/ml progranulin for 48 hours and then injected into NOG SCID gamma mice in serial dilution format. Xenograft results were calculated using Extreme Limiting Dilution Analysis (ELDA) software to determine the CSC frequency and significance.


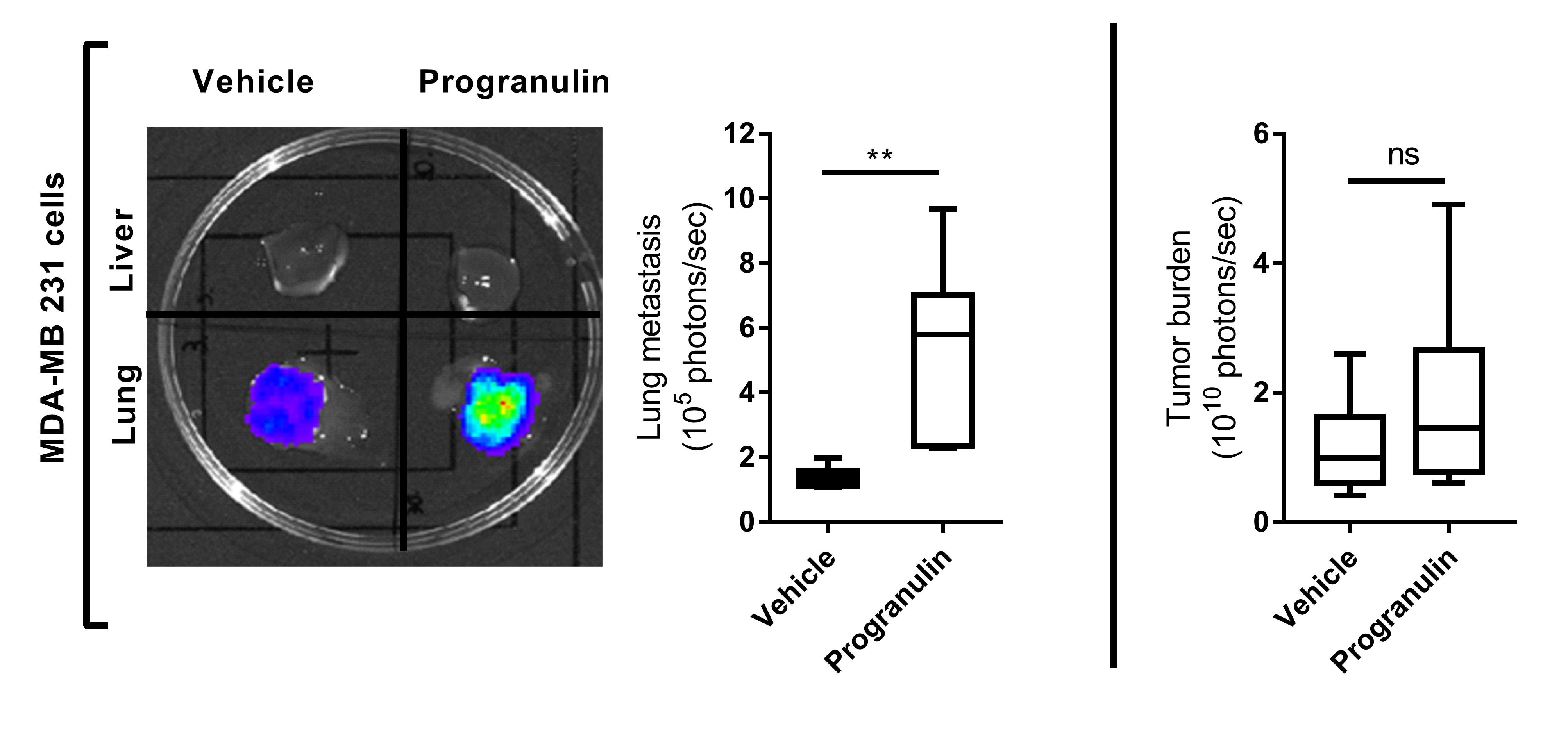


**Figure S2. Progranulin induces breast cancer metastases *in vivo***

MDA-MB 231 -luc xenografts where treated with either vehicle (PBS) or 8µg of progranulin three times per week for 6 weeks by subcutaneous injection. Tumour burden (right) and lung metastasis (left) luciferase measurements at the experimental endpoint are expressed as mean photons/second (right, top and bottom respectively) (n=6). Mann-Whitney u test was used for statistics. ** indicates p<0.01.


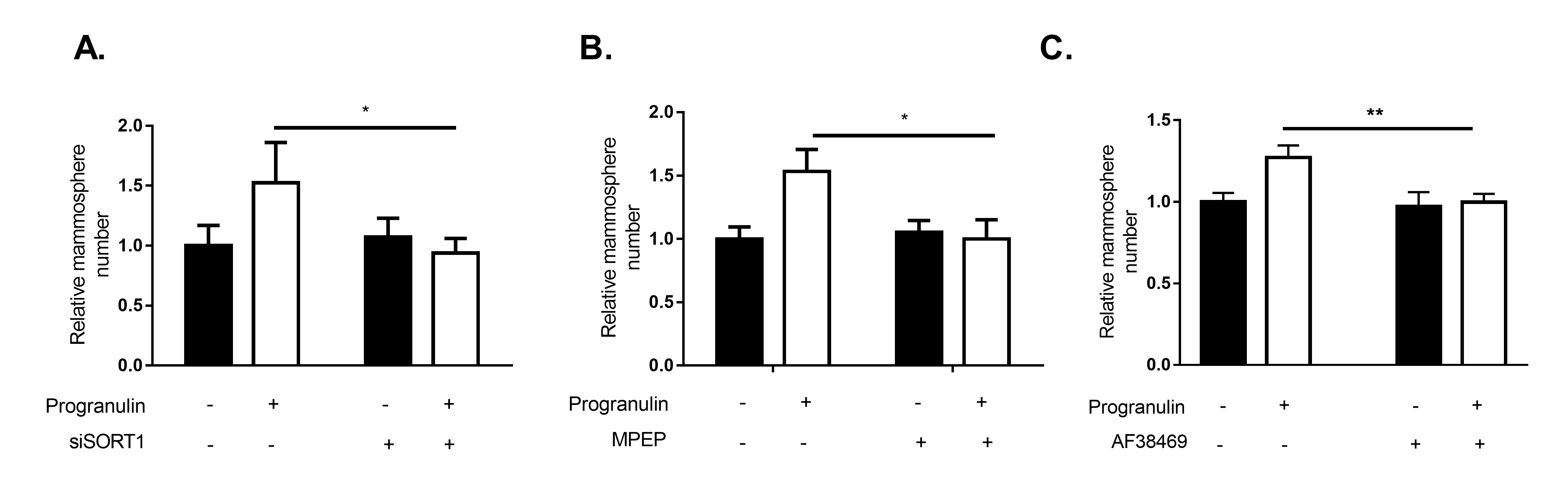


**Figure S3. The receptor sortilin is a prerequisite for progranulin induced CSC-like propagation.**

**(A)** MDA-MB 231 cells treated with siRNA against sortilin or scrambled control were pre-treated with either vehicle (PBS) or 1µg/ml progranulin and analysed for mammosphere forming capacity. Results are expressed as relative mammosphere number ±SD (n=3). (**B**) MDA-MB 231 cells treated with either vehicle or the sortilin degrading drug MPEP (10µM). 3 hours later, cells were treated with either vehicle (PBS) or 1µg/ml progranulin for 48 hours and analysed for mammosphere forming capacity. Results are expressed as relative mammosphere number ±SD (n=3) **(C)** MDA-MB 231 cells treated with either vehicle (PBS) or 1µg/ml progranulin with or without the small molecule AF38469 (10µM) and analysed for mammosphere forming capacity. Results are expressed as relative mammosphere number ±SD (n=3) * indicates p<0.05 and ** p<0.01 as calculated by a student’s t-test.

**
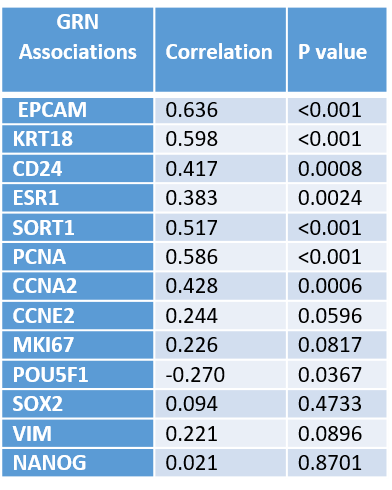

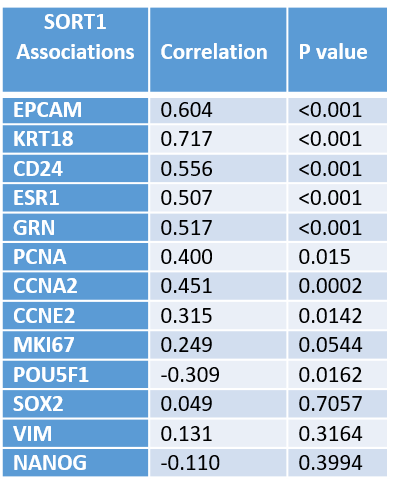
**

**Figure S4.** (left) *GRN* expression or (right) *SORT1* expression in MCF7 cells with selected functional transcripts. Correlation co-efficient is calculated by Spearman’s correlation and p-values are indicated in the figure.


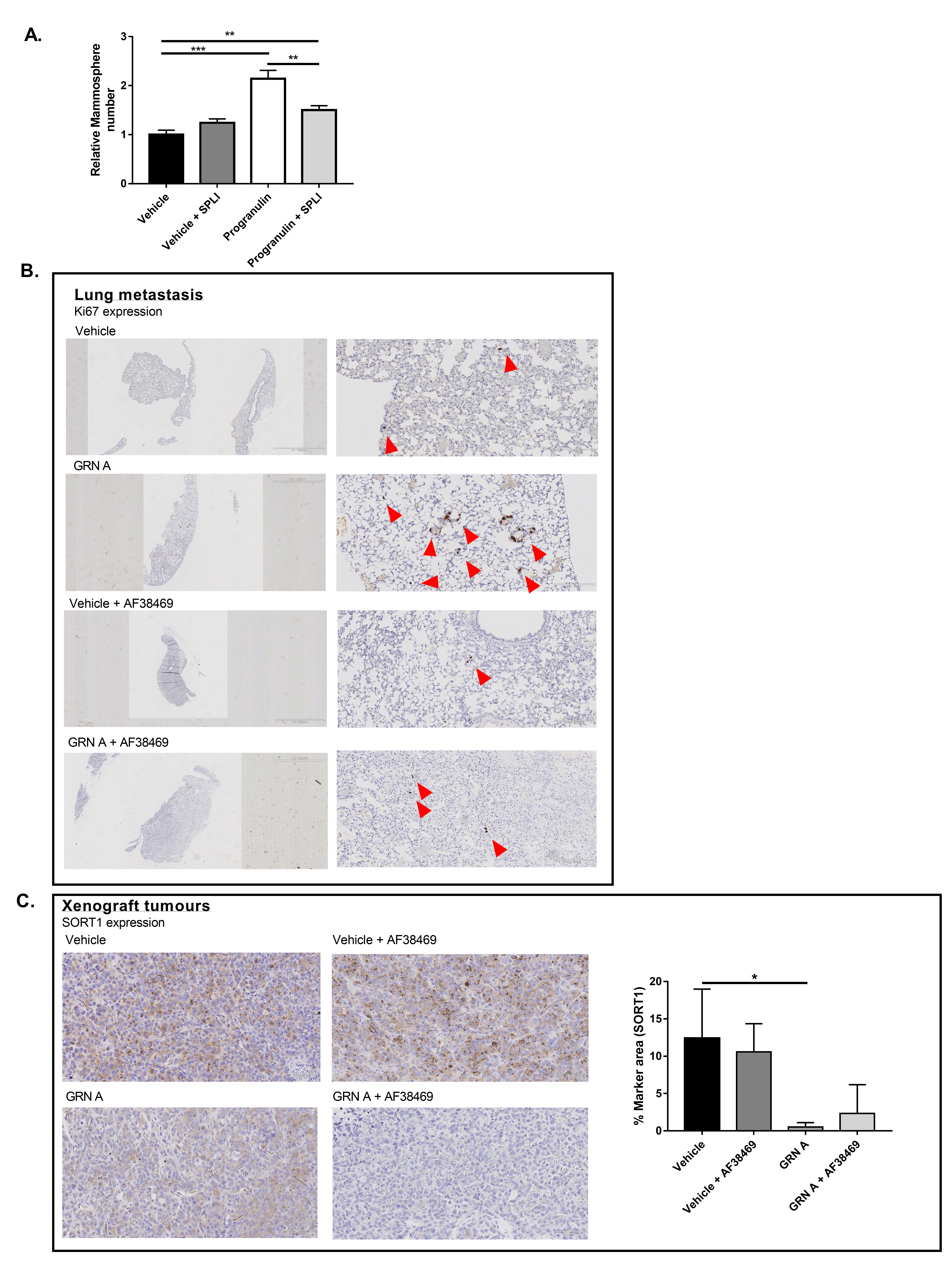


**Figure S5. The orally bioavailable small molecule AF38469 inhibit GRN domain A induced CSC-like propagation and disease metastasis *in vivo***

**(A)** MCF7 cells where treated with either vehicle (PBS) or 1µg/ml progranulin or in combination with 0,1µg/ml secretory leukocyte protease inhibitor (SPLI) and analysed for mammosphere forming capacity. Results are expressed as relative mammosphere number ±SD (n=3). ** indicates p<0.01 and *** *p*<0.001 as calculated by a student’s t-test. **(B)** Immunohistochemical analyses of the GRN A and AF38469 treated groups of MDA-MB 231 luc lung sections was performed using Ki67 and Haematoxylin staining. Arrows indicates infiltrating MDA-MB 231 luc cells tumour cells. Scale bar represents 1 x 2000 µm and 12 x 200 µm. **(C)** (left) Immunohistochemical analyses of the GRN A and AF38469 treated groups of MDA-MB 231 luc xenografts was performed using SORT1 and Haematoxylin staining. Scale bar represents 18 x 100 µm. **(C)** (right) Quantification of SORT1 expression in MDA-MB 231 luc xenografts. * indicates p<0.05 as calculated by a student’s t-test.

**Table S1.** Sequence list for primers used during qPCR analysis.

| **Gene** | **Accession number** | **Left Primer 5' - 3'** | **Right Primer 5' - 3'** |
| --- | --- | --- | --- |
| CCNA2 | NM_001237.4 | AAGACGAGACGGGTTGC | GGCTGTTTACTGTTTGCTTTCC |
| POU5F1 | NM_002701 | CGAAAGAGAAAGCGAACCAG | AACCACACTCGGACCACATC |
| NANOG | NM_024865 | CCTATGCCTGTGATTTGTGG | AAGTGGGTTGTTTGCCTTTG |
| SOX2 | NM_003106 | ACACCAATCCCATCCACACT | CCTCCCCAGGTTTTCTCTGT |
| VIM | [NM_003380.3](https://www.ncbi.nlm.nih.gov/nucleotide/240849334?report=genbank&log$=nucltop&blast_rank=2&RID=TN0NC0X5016) | CAGATGCGTGAAATGGAAGA | TGGAAGAGGCAGAGAAATCC |
| PCNA | [NM_182649.1](https://www.ncbi.nlm.nih.gov/nucleotide/33239450?report=genbank&log$=nucltop&blast_rank=1&RID=TN0Z25N1016) | GTGGAGAACTTGGAAATGGAA | ACCGTTGAAGAGAGTGGAGTG |
| MKI67 | [NM_001145966.1](https://www.ncbi.nlm.nih.gov/nucleotide/225543214?report=genbank&log$=nucltop&blast_rank=1&RID=TN168AHM016) | TGGGTCTGTTATTGATGAGCC | CATCAGGGTCAGAAGAGAAGC |
| CD24 | [NM_001291738.1](https://www.ncbi.nlm.nih.gov/nucleotide/619329007?report=genbank&log$=nucltop&blast_rank=1&RID=TN1A83JG014) | GCTCCTACCCACGCAGATT | GGTGGTGGCATTAGTTGGAT |
| EPCAM | NM_002354.2 | TGGTGTTATTGCTGTTATTGTGG | TCAGCCTTCTCATACTTTGCC |
| ESR1 | NM_000125.3 | TCCTGATGATTGGTCTCGTC | ATGCCCTCTACACATTTTCCC |
| SORT1 | [NM_002959.5](http://www.ncbi.nlm.nih.gov/nuccore/NM_002959.5) | ATGGGAAGAAATCCACAAAGCAG | ATTCCAGAGCCCCAAGGTCAG |
| GRN | [NM_002087.2](http://www.ncbi.nlm.nih.gov/nuccore/NM_002087.2) | CCAAAGATCAGGTAACAACTCCG | CATCGACCATAACACAGCACG |
| CCNE2 | NM_057749.2 | TCTCCTGGCTAAATCTCTTTCTCC | ACTGTCCCACTCCAAACCTG |
| KRT18 | NM_000224.2 | GCTGGAAGATGGCGAGGACTTT | TGGTCTCAGACACCACTTTGCC |
| PCNA | [NM_182649.1](https://www.ncbi.nlm.nih.gov/nucleotide/33239450?report=genbank&log$=nucltop&blast_rank=1&RID=TN1HBBWZ016) | GTGGAGAACTTGGAAATGGAA | ACCGTTGAAGAGAGTGGAGTG |
