# Supplementary figures and images for: Sortilin inhibition limits secretion-induced progranulin-dependent breast cancer progression and cancer stem cell expansion
Source: Breast Cancer Res. 2018 Nov 20;20:137. doi: 10.1186/s13058-018-1060-5 (PMC6245804; doi:10.1186/s13058-018-1060-5)

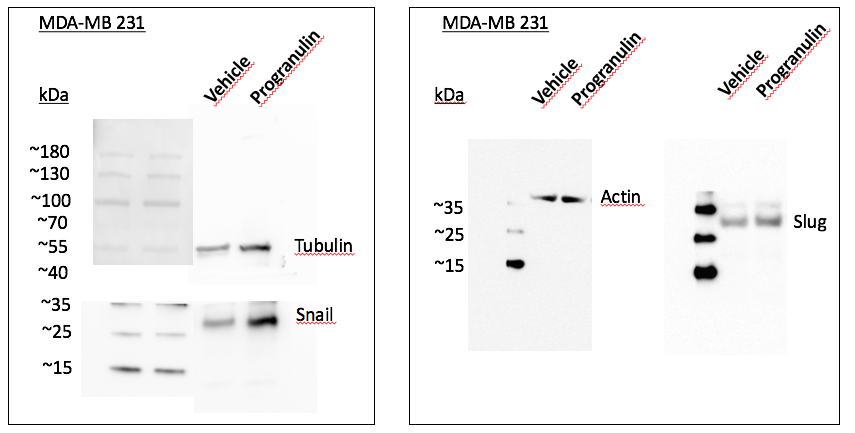

Supplement: Supplementary file 2 — Increase in protein expression of the transcription factors SNAIL (SNAI2) and SLUG (SNAI1) in progranulin-treated MDA-MB 231 breast cancer cells. MDA-MB 231 cells treated with either vehicle (PBS) or 1 μg/ml progranulin (×) and analysed for protein expression (Western blot). Left box illustrates protein ladder and targeted proteins at different exposure times. (TIFF 1453 kb) [file 13058_2018_1060_MOESM2_ESM.tiff]

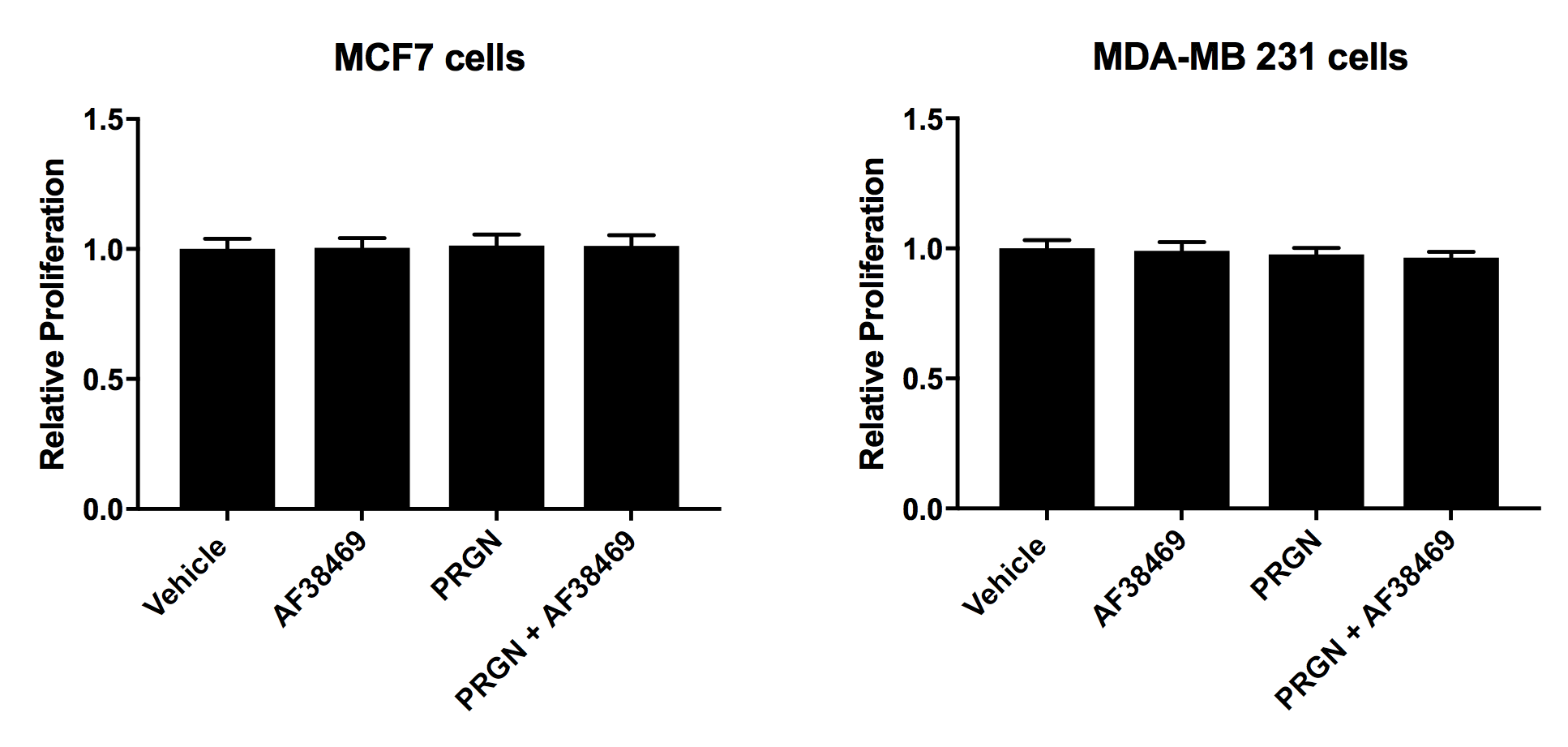

Supplement: Supplementary file 3 — The small orally available sortilin inhibitor AF38469 do not impact cell viability. Relative proliferation of MCF7 and MDA-MB 231 cells 48 h after treatment with vehicle (PBS/DMSO), AF38469 (3 μg/ml), progranulin (PGRN) (1 μg/ml) or AF38469 (3 μg/ml) and PGRN (1 μg/ml) by Alamar Blue assay. (TIFF 134 kb) [file 13058_2018_1060_MOESM3_ESM.tiff]

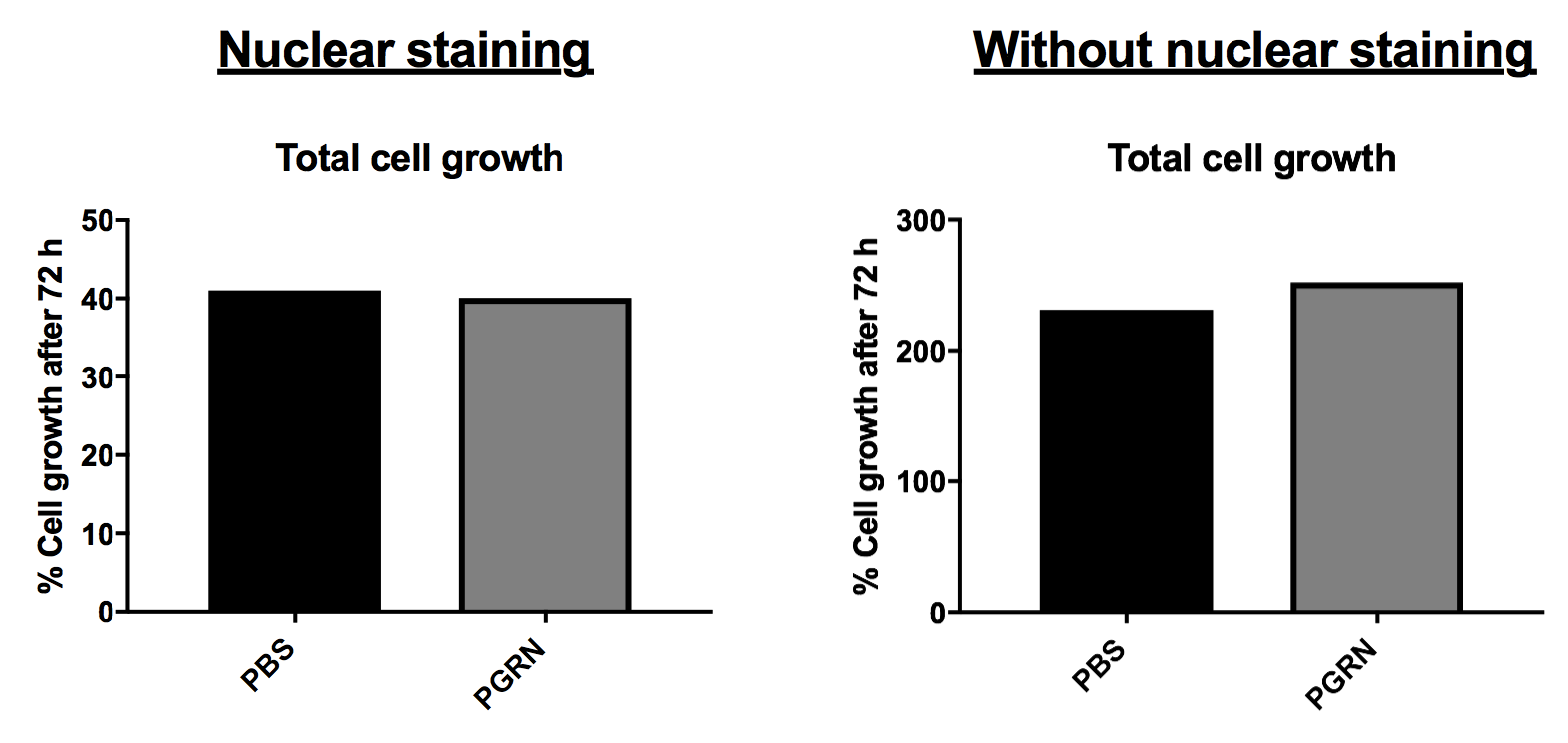

Supplement: Supplementary file 4 — Decreased proliferation of nuclear stained GFP-Sox2 reporter cells at 72 h of treatment. Percentage total cell growth of GFP-Sox2 reporter cells at 72 h of treatment with PBS or progranulin (PGRN) (1 μg/ml). Left bar plots demonstrate nuclear-stained cells with NucBlue and right bar plot demonstrate unstained GFP-Sox2 reporter cells. (TIFF 110 kb) [file 13058_2018_1060_MOESM4_ESM.tiff]
